# Relationships between measures of boat acceleration and performance in rowing, with and without controlling for stroke rate and power output

**DOI:** 10.1371/journal.pone.0249122

**Published:** 2021-08-20

**Authors:** Ana C. Holt, Kevin Ball, Rodney Siegel, William G. Hopkins, Robert J. Aughey

**Affiliations:** 1 Institute for Health and Sport (iHeS), Victoria University, Melbourne, VIC, Australia; 2 Australian Institute of Sport, Canberra, ACT, Australia; 3 Sport Science Department, Victorian Institute of Sport, Melbourne, VIC, Australia; University of Trás-os-Montes and Alto Douro, PORTUGAL

## Abstract

**Purpose:**

Boat acceleration profiles provide a valuable feedback tool by reflecting both rower technique and force application. Relationships between measures of boat acceleration and velocity to inform interpretation of boat acceleration profiles in rowing were investigated here.

**Methods:**

Thirteen male singles, nine female singles, eight male pairs, and seven female pairs participated (national and international level, age 18–27 y). Data from each stroke for 74 2000-m races were collected using Peach PowerLine and OptimEye S5 GPS units. General linear mixed modelling established modifying effects on velocity of two within-crew SD of boat acceleration variables for each boat class, without and with adjustment for stroke rate and power, to identify potential performance-enhancement strategies for a given stroke rate and power. Measures of acceleration magnitude at six peaks or dips, and six measures of the rate of change (jerk) between these peaks and dips were analyzed. Results were interpreted using rejection of non-substantial and substantial hypotheses with a smallest substantial change in velocity of 0.3%.

**Results:**

Several boat acceleration measures had decisively substantial effects (-2.4–2.5%) before adjustment for stroke rate and power. Most effect magnitudes reduced after adjustment for stroke rate and power, although maximum negative drive acceleration, peak drive acceleration, jerk during the mid-drive phase, and jerk in the late recovery remained decisively substantial (-1.8–1.9%) in some boat classes.

**Conclusion:**

Greater absolute values of maximum negative drive acceleration and jerk in the late recovery are related to improved performance, likely reflecting delayed rower centre-of-mass negative acceleration in preparation for the catch. Greater absolute values of peak drive acceleration, first peak acceleration, and jerk in the early and mid-drive are also associated with improved performance, likely reflecting propulsive force during the drive. These proposed mechanisms provide potential strategies for performance enhancement additional to increases in stroke rate and power output.

## Introduction

Boat acceleration profiles provide insight into rower force application and centre-of-mass (COM) movement, and are frequently used by coaches and sport scientists to provide feedback on rowing technique. Rower COM acceleration occurs in the direction of boat travel during the drive (propulsive) phase and is reversed during the recovery (non-propulsive) phase, contributing to fluctuations in boat acceleration throughout the stroke [1]. Discontinuous force application throughout the stroke, and the timing of force application with rower COM acceleration further contributes to boat acceleration fluctuations [1, 2], with the acceleration profile closely reflecting force curve shape during the drive [3]. Boat acceleration profiles have been used for several years by coaches and sport scientists as a method of biomechanical and technical analysis. However, current interpretation of acceleration profiles is often informed by the comparison of profiles to those produced by successful rowers, given few studies have investigated relationships between the acceleration profile and rowing performance [1, 4, 5].

**Fig 1:**
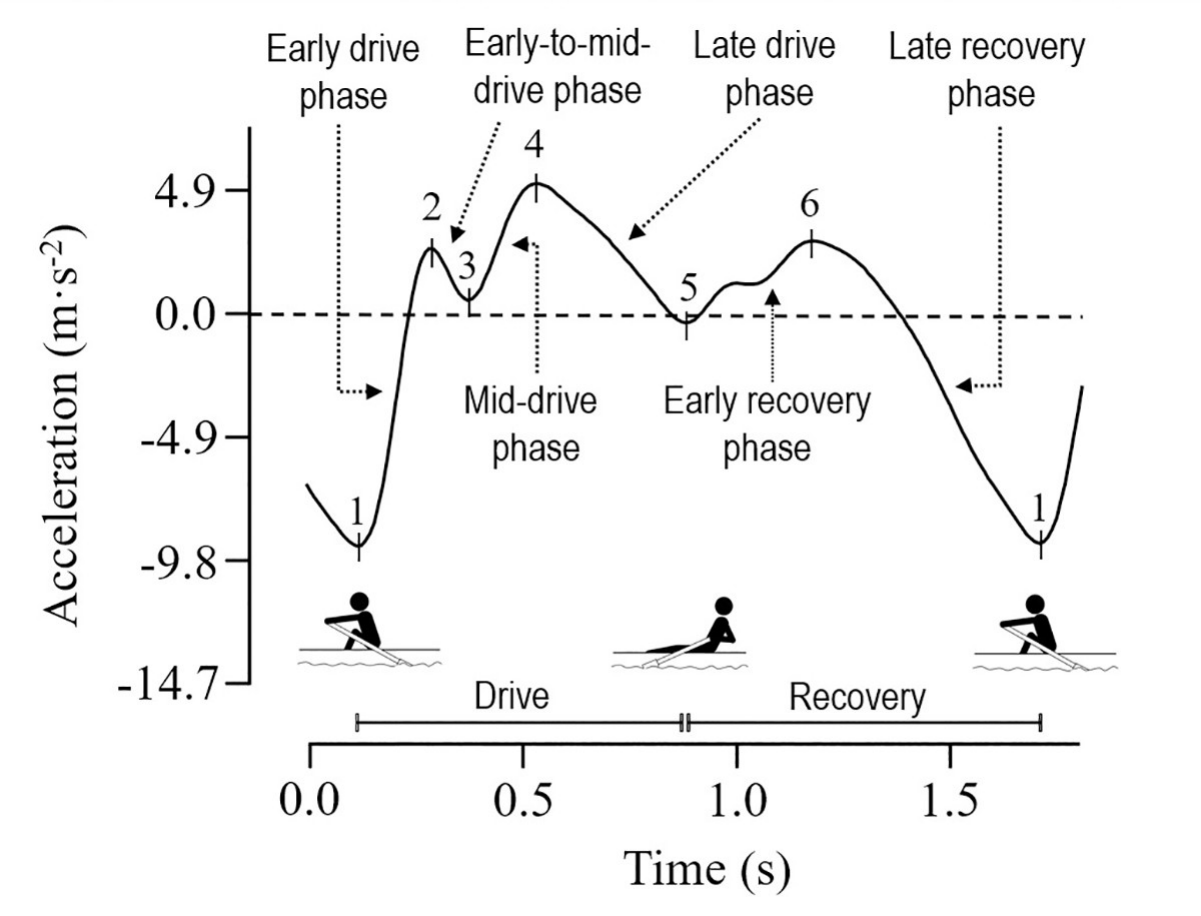
Filtered boat acceleration over a single stroke in a men’s coxless pair at 37.5 strokes·min^-1^. 1, maximum negative drive acceleration and the approximate start of drive and end of recovery phases; 2, first peak; 3, first dip; 4, peak drive acceleration; 5, finish dip and the approximate end of drive and start of recovery phases; 6, peak recovery acceleration. The respective phases for the analysis of jerk are: Early drive phase, markers 1 to 2; early-to-mid drive phase, markers 2 to 3; mid-drive phase, markers 3 to 4; late drive phase, markers 4 to 5; early recovery phase, markers 5 to 6; late recovery phase, marker 6 to 1 of the next stroke. Rower position corresponding to the acceleration profile is shown above.

Research investigating relationships between boat acceleration and rowing performance is mostly limited to the assessment of men’s pairs, often with small sample sizes, and involves the comparison of crews with varying success levels [1, 4–6], adding to the difficulty faced by coaches and sport scientists in identifying favourable measures of boat acceleration. Conflicting positive [4] and negative [1, 5] relationships with rowing success level have been observed for the magnitude of maximum negative acceleration occurring early in the drive (marker 1 in Fig 1) in elite men’s pairs. Greater magnitudes of maximum negative drive acceleration occur in sweep (one oar per rower) compared to sculling (two oars per rower), and male compared to female boat classes [6]. Jerk, the rate of acceleration change [7] is greater following maximum negative drive acceleration (markers 1 to 2 in Fig 1) in more successful crews, as is an earlier occurrence of positive boat acceleration [1, 5]. Acceleration magnitude at the first positive peak of acceleration occurring during the drive (marker 2 in Fig 1) is positively associated with rowing success level [1], with larger magnitudes in sweep compared to sculling boat classes [6]. The subsequent dip in acceleration following the first peak (marker 3 in Fig 1) also has a positive association with crew success level [1]. Negative associations exist between peak drive acceleration magnitude (marker 4 in Fig 1) and crew success [5]. Nevertheless, the small sample sizes and somewhat conflicting results of these studies make inferences regarding favourable acceleration profiles difficult. Furthermore, only a small section of the acceleration profile (up to marker 4 in Fig 1) has been examined, whereby the association between boat acceleration in the recovery phase and rowing performance is not known.

Further research investigating the boat acceleration profile across the whole stroke in detail, and its relationship with rowing performance, would better inform how rower COM movement and force application impact rowing performance, and therefore better inform the interpretation of boat acceleration profiles in rowing. Given the magnitudes of acceleration at the first positive peak and the maximum negative drive acceleration are related to stroke rate [1], research investigating the boat acceleration profile when adjusting for stroke rate would ensure any relationships between boat acceleration and performance are not simply a product of higher stroke rates, and inform potential strategies for enhancing performance in addition to increases in stroke rate. Similarly, adjusting for power output when assessing the relationship between boat acceleration and performance would reveal performance effects that are related to improved rowing efficiency rather than the power applied. Therefore, this study aims to inform the interpretation of the boat acceleration profile in rowing by investigating the effect of the boat acceleration profile on boat velocity in men’s and women’s singles and coxless pair boats during 2000-m racing without and with adjustment for stroke rate and power. The outcomes of this investigation will inform which aspects of the boat acceleration profile correspond with the greatest improvements in rowing performance, and how these relationships are mediated by stroke rate and power output, advising areas where additional performance improvements can be achieved.

## Materials and methods

### Participants

Twenty-one female (age 20.6 ± 2.2 y; height 176.6 ± 6.2 cm; body mass 72.5 ± 7.9 kg) and 23 male (age 21.0 ± 2.5 y; height 189.5 ± 8.0 cm; body mass 85.8 ± 9.7 kg) national and international-level rowers who performed regular training volumes of approximately 17–22 h·wk^-1^ volunteered for this study. Participants provided informed consent prior to commencement of the study. The study was approved by the Victoria University Human Research Ethics Committee (VUHREC; approval number HRE19-036), and written consent was obtained from participants.

### Study design

The study was conducted during three national regattas held at the Sydney International Regatta Centre, Australia. A total of 74 2000-m races were recorded from 14 male single crews (25 races), nine female single crews (18 races), nine male coxless pair crews (18 races), and seven female coxless pair crews (13 races). The data collected includes that used in Holt et al. [8] for the analysis of technical determinants of rowing performance, with the inclusion of data collected from 27 additional races in the current study. Of the men’s singles crews, six competed in lightweight events (10 races), as did one of the female single crews (two races). Crew age categories were <19 y (one crew), <21 y (seven crews), <23 y (24 crews), and Senior (no age restriction; seven crews). Races recorded were heats (32 races), repecharges (four races), semi-finals (eight races) and finals (30 races). The number of races analysed ranged from one to five for any given crew; these repeated measurements were accounted for in the mixed model, as described in the statistical analyses section. One participant in the men’s single and one in the women’s single also competed in the coxless pair, and three male and one female participant competed in two coxless pair crews (i.e., with a different pair partner, assessed as separate crews). Crews were given no instructions from the researchers regarding race strategy or stroke rate. Power output was collected per stroke from races using Peach PowerLine instrumentation systems (Peach Innovations, UK), calibration of force and gate angle was performed immediately prior to each race. Boat velocity and acceleration was collected at a sample rate of 10 Hz and 100 Hz respectively, using OptimEye S5 GPS units (Catapult, Australia) attached to the stern canvas of participant boats. Both Peach PowerLine instrumentation and Catapult GPS systems are used frequently within elite rowing programs. Acceptable levels of validity have been established for measures of rowing velocity from Catapult GPS units (0.2% standard error of the estimate) [9] and for force and oar angle (<8.9 N and <0.9° standard error of the estimate, respectively) by Peach instrumentation systems [10]. The Peach system calculates power from measures of gate angular velocity, gate force in the direction of the boat’s long axis, and the ratio of the oar outboard (distance from the collar to blade tip) to total length. Power from the Peach system represents a proxy measure of the true mechanical power output [11]. Venue environmental conditions (collected at 1-min intervals from six weather stations positioned at water level along the 2000 m course) were: 21.9 ± 2.4°C air temperature (mean ± SD); 26.0 ± 1.2°C water temperature; 70.5 ± 20.4% relative humidity; and 1.3 ± 0.5 m·s^-1^ wind speed, in a predominantly cross direction on stroke side (port).

### Data processing

Acceleration and velocity data was exported from the software Logan (version 48.41, Australian Institute of Sport, Australia) and processed in the desktop version of R Studio (version 1.2.5, R Foundation, Austria). A low-pass 4^th^ order Butterworth filter with 6 Hz cut-off frequency was applied to acceleration data (the choice of cut-off frequency was based on residual analysis and visual inspection of raw and smoothed curves).

**Fig 2:**
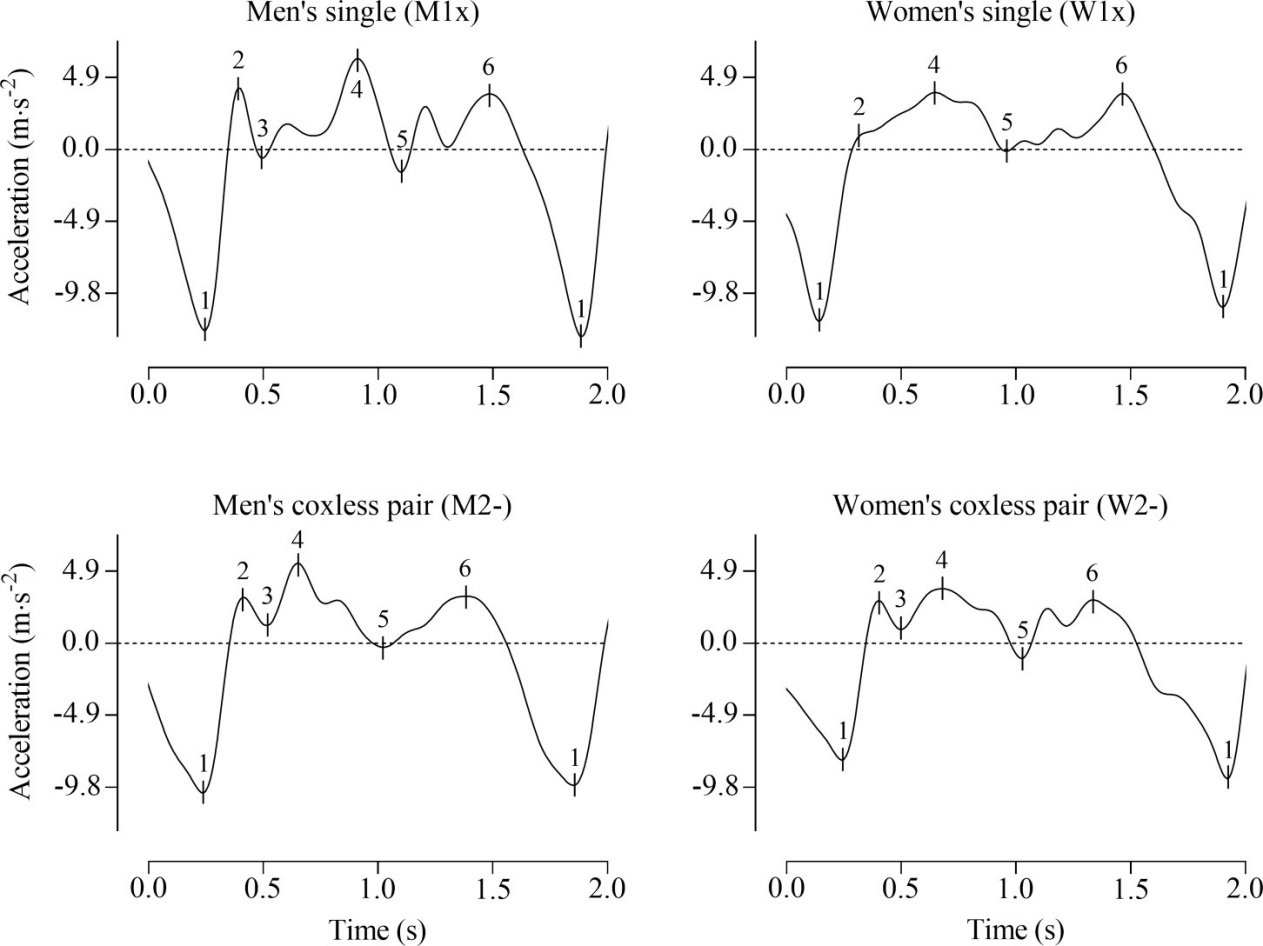
Filtered boat acceleration over a single stroke from a crew in each of the four boat classes. 1, maximum negative drive acceleration; 2, first peak; 3, first dip; 4, peak drive acceleration; 5, finish dip; 6, peak recovery acceleration. Strokes rates for the four profiles are: 36.6 strokes·min^-1^, men’s singles; 33.9 strokes·min^-1^, women’s singles; 37.5 strokes·min^-1^, men’s coxless pairs; 35.9 strokes·min^-1^, women’s coxless pairs.

Three peaks and three dips in acceleration magnitude were identified in each stroke, as shown in the acceleration profile of a single stroke in a men’s coxless pair crew in Fig 1, and from a crew in each of the four boat classes in Fig 2. The variable maximum negative drive acceleration was the largest negative acceleration magnitude, and was used to define the start and end of each stroke (marker 1 in Fig 1). The variable peak drive acceleration was the largest positive acceleration occurring between 25 to 66% of total stroke duration (marker 4 in Fig 1; this range ensured the first peak and finish peak were not identified as this variable; as such, if the absolute maximal peak acceleration during the drive phase occurred at the first peak it was not marked as the peak drive acceleration variable, and the magnitude of the peak drive acceleration variable was therefore not the true maximum in acceleration during the drive phase). The variable peak recovery acceleration was the largest positive acceleration occurring later than 0.3 s after the peak drive acceleration variable (marker 6 in Fig 1; the 0.3 s delay ensured the point identified occurred in the recovery phase and was not a subsequent acceleration peak occurring in the drive phase). The finish dip variable was marked as the first occurrence after the location of the peak drive acceleration variable where acceleration increased and was less than 2.45 m·s^-2^ (marker 5 in Fig 1; the 2.45 m·s^-2^ threshold ensured any acceleration dips occurring during the drive phase after the peak drive acceleration variable were not marked as the finish dip variable). The first acceleration peak variable was the first occurrence after the maximum negative drive acceleration of a jerk of less than 0.20 m·s^-3^, where acceleration was greater than -0.98 m·s^-2^ (marker 2 in Fig 1; these thresholds allowed identification of a plateau in acceleration, or a reduction in jerk that occurred early in the drive when no peak occurred, such as that at marker 2 in Fig 2 for the Women’s single). A reduction in jerk was identified in the absence of a peak for the first acceleration peak variable, as shown at marker 2 in Fig 2 for the Women’s single, in approximately 7% of the strokes analysed. The first acceleration dip variable was the first increase in acceleration that occurred following the first peak variable (marker 3 in Fig 1). For strokes where a reduction in jerk or plateau in acceleration were identified in the absence of a peak for the first peak variable, the first dip variable was excluded from analyses as it did not occur either (marker 2 in Fig 2 for the Women’s single). For the purposes of describing the stroke in this study, the drive phase was defined as between maximum negative drive acceleration and the finish dip, and the recovery phase was defined as between the finish dip and maximum negative drive acceleration of the following stroke (Fig 1).

Jerk, the rate of acceleration change (m·s^-3^), was calculated from the absolute change in acceleration over the phase duration, for six phases per stroke using the three peak and dips in acceleration magnitude defined above (Fig 1). The six phases were: early drive (between maximum negative drive acceleration and the first peak in the early drive); early-to-mid-drive (between the first peak and the first dip); mid-drive (between the first dip and peak drive acceleration); late drive (between peak drive acceleration and finish dip); early recovery (between finish dip and peak recovery acceleration); and late recovery (between peak recovery acceleration and maximum negative drive acceleration of the next stroke). Where the first dip did not occur (i.e. acceleration did not decrease or plateau following the first peak) jerk in the mid-drive was calculated between the first peak and peak drive acceleration.

Stroke rate (strokes·min^-1^) was calculated from the duration between the start and end of each stroke. Velocity per stroke (m·s^-1^) was the mean velocity between the start and end of each stroke. Power (W) per stroke was exported from Peach units and aligned from the first stroke of the race with acceleration outputs per stroke in R studio. Peach units use gate angle velocity, gate force in the direction of the boat’s long axis, and the oar outboard (distance from the collar to blade tip)-to-total length ratio to calculate power.

The first ten strokes of each race were excluded from analyses in order to assess strokes where boat velocity was reasonably consistent, given these strokes encompass an initial acceleration phase of the boat from a stationary starting position and include partial strokes that are not representative of a typical rowing stroke. The last ten strokes were excluded from analyses, given that large changes in velocity can occur at the end of the race, such as a sprint to the line involving partial strokes to gain a desired a finishing position, or a substantial decrease in velocity and stroke rate where the race outcome is already secured. Outlier stroke values for each predictor variable were identified as those with a standardized difference from the mean greater than 4.5 [12], where the mean and the standardizing SD were the running mean and running standard deviation for up to 30 strokes preceding and up to 30 strokes following the given stroke (depending on the location of the stroke in the race). A second pass of the running mean and running standard deviation was used to eliminate visually obvious outliers that were missed in the first pass. Additional outliers were identified as strokes with a standardized residual greater than 4.5 after running the statistical analyses [12]. All outliers identified were excluded from analysis, and ranged from 0.4% to 0.9% of the total strokes recorded per boat class.

### Statistical analysis

Each gender and boat class was analysed separately with the general linear mixed-model procedure (Proc Mixed) in the Studio University edition of the Statistical Analysis System (version 9.4, SAS Institute, Cary NC). The modelling was similar to that in Holt et al. [8], who performed a stroke-to-stroke analysis of the effect of biomechanical measures of rowing technique on boat velocity. The mean modifying effect on velocity of a two standard deviation (SD) within-crew change of each predictor (described above and presented in Table 1) was estimated from the values for each predictor per stroke and the corresponding mean boat velocity per stroke. Mean boat velocity was taken from the next stroke for the analysis of jerk in the late recovery, as this variable occurred late in the stroke and is expected to effect the velocity of the following stroke. The fixed effects in the model, predicting the logarithm of boat velocity (V), were each predictor variable analysed separately as linear predictors. Separate analyses were conducted to adjust for stroke rate, and to adjust for power output (P, the sum of both oars) and stroke rate. Fixed effects in these models were log(stroke rate) (adjusting log(V) for stroke rate), and log(P) and log(stroke rate) (adjusting log(V) for power output and stroke rate), and each predictor separately as linear predictors. In these analyses log(V) predicted by log(P) allows estimation of k and x in the kinetic equation V = k·P^x^. Random effects in the model were: crew identity (to adjust for consistently better or worse velocity of each crew across all races); crew identity interacted with log(P) (representing individual differences in the exponent x, and allowing for this term and crew identity to be correlated via an unstructured covariance matrix); the given predictor interacted with crew identity (to estimate individual differences between crews in the effect of the variable, and to account for differences in the number of repeated measurements [i.e., races analysed] between crews); race identity (to adjust for between-race changes in mean velocity due to changes in environmental conditions [such as wind and temperature] and the efficiency of the crew); and a different residual error for each crew (representing stroke-to-stroke variability in velocity [e.g., due to wind gusts or the blade catching water on the recovery] not accounted for by the other effects). The random effects for race identity and the different residuals for crews account for environmental effects, which therefore do not contribute directly to the effects of acceleration variables on boat velocity.

**Table 1:** Characteristics of boat velocity, stroke rate, and power, and the predictor variables in the four boat classes.

	Singles		Coxless pairs	
	Men (M1x)	Women (W1x)	Men (M2-)	Women (W2-)
Boat velocity (m·s^-1^)	4.60 ± 0.10/0.19	4.13 ± 0.09/0.25	4.88 ± 0.10/0.25	4.34 ± 0.13/0.21
Stroke rate (strokes·min^-1^)	35.1 ± 1.8/1.9	32.5 ± 1.1/1.7	37.2 ± 1.2/1.9	35.5 ± 1.8/1.9
Boat power (W)^b^	337 ± 38/34	221 ± 21/24	704 ± 60/88	481 ± 40/59
**Acceleration magnitude**				
Max negative drive (m·s^-2^)	-11.8 ± 1.4/1.2	-10.3 ± 1.6/0.9	-11.1 ± 1.0/1.3	-9.02 ± 1.4/1.1
First peak (m·s^-2^)	2.35 ± 0.98/0.59	2.55 ± 0.98/0.49	2.84 ± 0.69/0.69	2.26 ± 0.78/0.69
First dip (m·s^-2^)	0.20 ± 0.98/0.39	0.69 ± 0.69/0.29	1.18 ± 0.78/0.39	0.69 ± 0.49/0.39
Peak drive (m·s^-2^)	5.20 ± 0.78/0.39	3.82 ± 0.39/0.29	5.39 ± 0.29/0.49	3.92 ± 0.39/0.39
Finish dip (m·s^-2^)	-0.59 ± 0.39/0.29	-0.59 ± 0.29/0.20	-0.29 ± 0.39/0.29	-0.59 ± 0.29/0.20
Peak recovery (m·s^-2^)	3.04 ± 0.78/0.39	2.75 ± 0.69/0.29	3.24 ± 0.49/0.39	2.94 ± 0.39/0.39
**Jerk**				
Early drive (m·s^-3^)	83 ± 15/13	72 ± 18/8.8	76 ± 10/14	62 ± 8.8/13
Early-to-mid-drive (m·s^-3^)	-15 ± 12/7.8	-22 ± 11/4.9	-19.6 ± 7.8/7.8	-18.6 ± 4.9/6.9
Mid-drive (m·s^-3^)	15.7 ± 7.8/2.9	8.8 ± 3.9/2.0	26.5 ± 3.9/6.9	15.7 ± 3.9/6.9
Late drive (m·s^-3^)	-29.4 ± 1.2/3.9	-18.6 ± 8.8/2.9	-17.7 ± 2.9/2.0	-15.7 ± 6.9/2.0
Early recovery (m·s^-3^)	7.8 ± 2.9/2.0	7.8 ± 2.0/2.0	10.8 ± 2.0/2.0	9.8 ± 2.9/2.0
Late recovery (m·s^-3^)	-35.3 ± 6.9/5.9	-27.5 ± 5.9/3.9	-29.4 ± 3.9/4.9	-23.5 ± 4.9/3.9

Data are mean ± between-crew SD/within-crew SD^a^.

M1x, men’s singles; W1x, women’s singles; M2-, men’s coxless pairs; W2- women’s coxless pairs; Max, maximum.

^a^Mean is the mean of the crew means, between-crew SD is the SD of the crew means, and within-crew SD is the mean of the crews’ SDs across their 1–3 races (~250 to ~750 strokes).

^b^Boat power is the sum of power from both oars.

Number of crews: 14, 9, 9 and 7 respectively.

Number of races: 25, 18, 18, 13 respectively.

A smallest substantial change in velocity of 0.3% was assumed, given the 1.0% race-to-race variation in 2000-m race times of elite rowers [13]. Corresponding magnitude thresholds for changes in velocity were: <0.3% trivial, ≥0.3% small, ≥0.9% moderate, ≥1.6% large, ≥2.5% very large, and ≥4.1% (≤-3.9% for negative effects) extremely large [12]. To evaluate magnitudes of SDs representing between-crew differences the magnitude thresholds are one-half of those in the above scales: <0.15% trivial, ≥0.15% small, ≥0.45% moderate, ≥0.8% large, ≥1.3% very large, and ≥2.0% extremely large [13].

Sampling uncertainty in the estimates of effects is presented as 90% compatibility limits. Decisions about magnitudes accounting for the uncertainty were based on one-sided interval hypothesis tests, where an hypothesis of a given magnitude (substantial, non-substantial) was rejected if the 90% compatibility interval fell outside that magnitude [14]. P-values for the tests were the areas of the sampling distribution of the effect (t for means, z for variances) falling in the hypothesized magnitude, with the distribution centred on the observed effect. Hypotheses of inferiority (substantial negative) and superiority (substantial positive) were rejected if their respective p-values (p_−_and p_+_) were <0.05; rejection of both hypotheses represents a decisively trivial effect in equivalence testing. When only one hypothesis was rejected, the p-value for the other hypothesis, when >0.25, was interpreted as the posterior probability of a substantial true magnitude of the effect in a reference-Bayesian analysis with a minimally informative prior [15] using the following scale: >0.25, possibly; >0.75, likely; >0.95, very likely; >0.995, most likely [12]; the probability of a trivial true magnitude (1 –p_–_−p_+_) was also interpreted, when >0.25, with the same scale. Probabilities were not interpreted for effects with inadequate precision at the 90% level, defined by failure to reject both hypotheses (p_–_>0.05 and p_+_>0.05). Effects with adequate precision at the 99% level (p_–_<0.005 or p_+_<0.005) are shown in bold in Supporting information tables, and represent effects that have a conservative low risk of harm (association with reduced velocities). The hypothesis of non-inferiority (non-substantial-negative) or non-superiority (non-substantial-positive) was rejected if its p value (p_N−_ = 1 –p_−_or p_N+_ = 1 –p_+_) was <0.05, representing a decisively substantial effect in minimal-effects testing: very likely or most likely substantial.

## Results

Mean values for predictor variables with between-crew and within-crew SD are presented in Table 1. Within-crew SD indicate half of the range that effects for predictors were assessed over.

**Fig 3:**
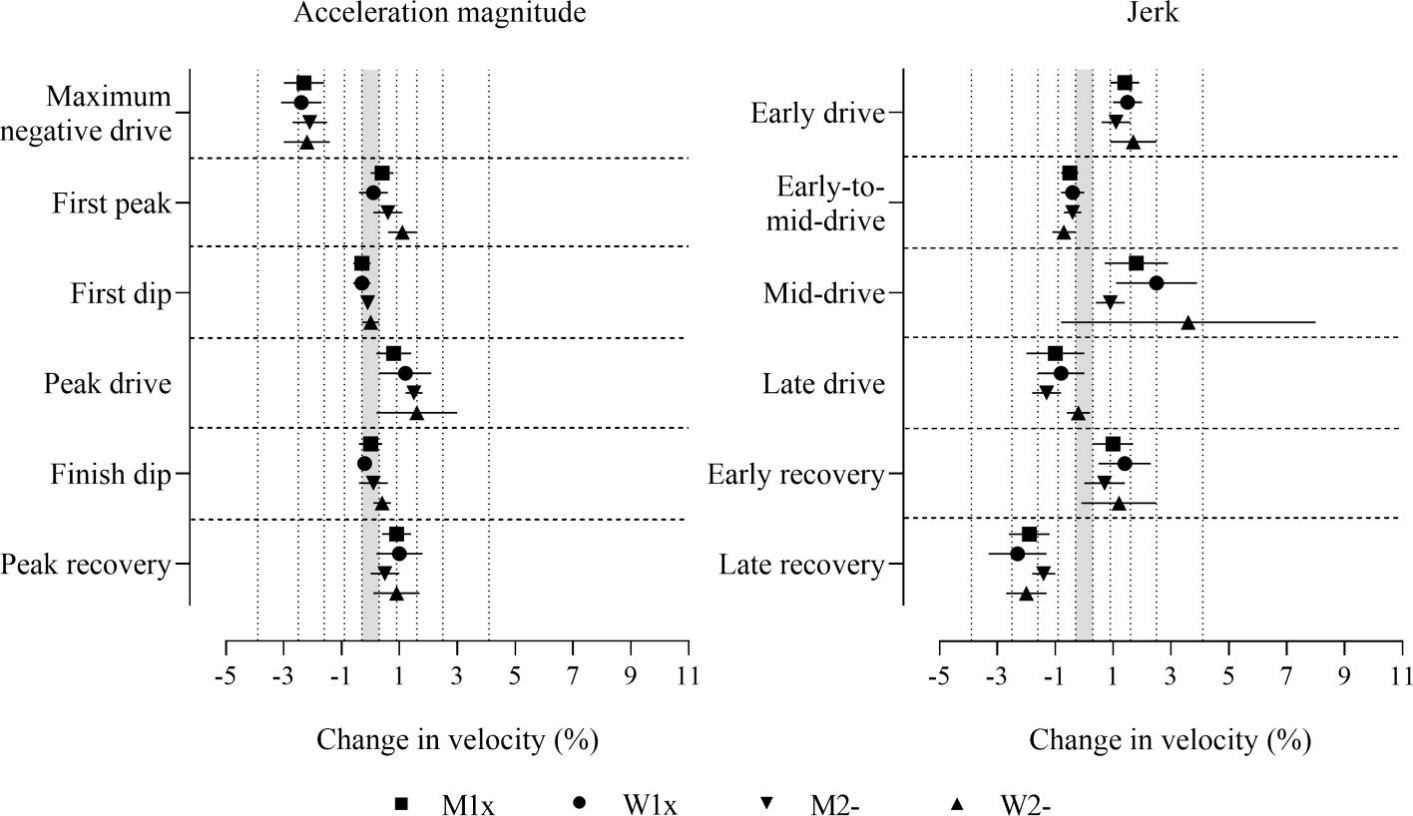
Change in boat velocity for a change in each predictor variable of two within-crew standard deviations without adjustment for stroke rate or power output. Data are mean (%) and 90% compatibility intervals. The shaded grey area covers trivial values (values within the smallest substantial change thresholds, -0.3 to 0.3%). Vertical dotted lines delineate threshold magnitudes of small (±0.3), moderate (±0.9), large (±1.6), very large (±2.5), and extremely large (4.1, -3.9%). Effects with compatibility intervals that do not enter the grey area are decisively (very likely or most likely) substantial. Effects with compatibility intervals that end within the grey area have adequate precision (possibly or likely substantial or trivial).

Before adjustment, consistent positive effects for jerk in the early drive, consistent negative effects for jerk in the late recovery and maximum negative drive acceleration magnitude were found in all boat classes, which were decisively substantial (Fig 3 and S1 Table). Positive effects for jerk in the mid-drive and early recovery, first dip, peak drive, and peak recovery acceleration magnitudes, and negative effects for jerk in the early drive and late drive were found in some boat classes (those with 90% compatibility intervals entirely in substantial values in Fig 3), which were decisively substantial. Measures that had adequate precision and observed substantial effects that were only possibly or likely substantial (only one of the superiority and inferiority hypotheses was rejected, p_+_ or p_−_>0.05), include positive effects in some boat classes for jerk in the early recovery, first peak, peak drive, and peak recovery acceleration magnitudes, and negative effects in some boat classes for jerk in the early-to-mid-drive and the late drive, first dip and finish dip acceleration magnitudes (those with more than half of the 90% compatibility intervals overlapping substantial values in Fig 3). Only one effect was decisively trivial, where both the superiority and inferiority hypotheses were rejected (p_+_ and p_−_<0.05), in men’s pairs for first dip acceleration magnitude. Trivial observed effects that had adequate precision but were only possibly or likely trivial were found in some boat classes for jerk in the late drive, first dip and finish dip acceleration magnitudes (those with less than half of the 90% compatibility intervals overlapping substantial values in Fig 3).

**Fig 4:**
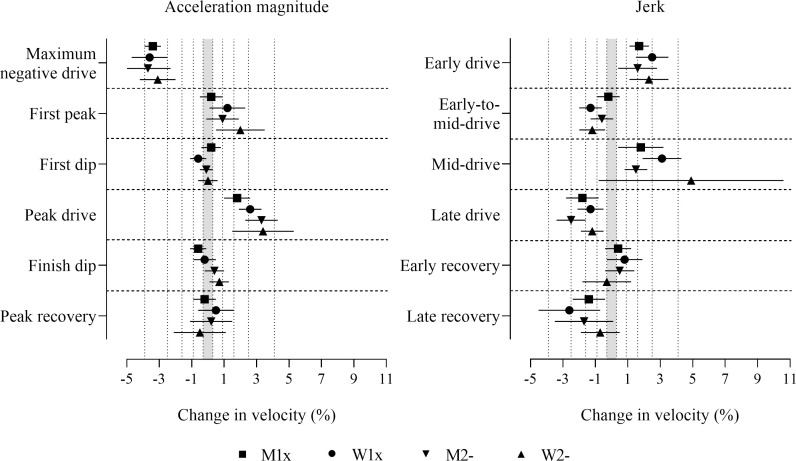
Change in boat velocity for a change in predictor variables of two within-crew standard deviations with adjustment for stroke rate. Data are mean (%) and 90% compatibility intervals. The shaded grey area covers trivial values (values within the smallest substantial change thresholds, -0.3 to 0.3%). Vertical dotted lines delineate threshold magnitudes of small (±0.3), moderate (±0.9), large (±1.6), very large (±2.5), and extremely large (4.1, -3.9%). Effects with compatibility intervals that do not enter the grey area are decisively (very likely or most likely) substantial. Effects with compatibility intervals that end within the grey area have adequate precision (possibly or likely substantial or trivial).

After the adjustment for stroke rate, effect magnitudes increased for jerk in the late drive, maximum negative drive, and peak drive acceleration magnitudes across all boat classes, and in some boat classes for jerk in the early-to-mid-drive and the late recovery, first peak and finish dip acceleration magnitudes (as shown in Fig 4 and S2 Table). Effect magnitudes decreased after adjustment for stroke rate for recovery peak acceleration magnitude in all boat classes, and for jerk in the early drive, the early recovery and the late recovery in some boat classes (refer to Fig 4 and S2 Table). Positive effects for peak drive acceleration, and negative effects for jerk in the late drive and maximum negative drive acceleration were decisively substantial in all boat classes. Positive effects for jerk in the early drive and the mid-drive, and first peak acceleration, and negative effects for jerk in the early-to-mid-drive) and the late recovery were decisively substantial in some boat classes (those with 90% compatibility intervals entirely in substantial values in Fig 4). Measures where the effects had adequate precision but were only possibly or likely substantial include positive effects in some boat classes for jerk in the early recovery and first peak acceleration magnitude, and negative effects in some boat classes for jerk in early-to-mid-drive and the late recovery, first dip and finish dip acceleration magnitudes (those with more than half of the 90% compatibility intervals overlapping substantial values in Fig 4). Precision was inadequate (the superiority and inferiority hypothesis were not rejected, p_+_ and p_−_>0.05) in most boat classes for jerk in the early recovery, first dip and peak recovery acceleration magnitudes.

**Fig 5:**
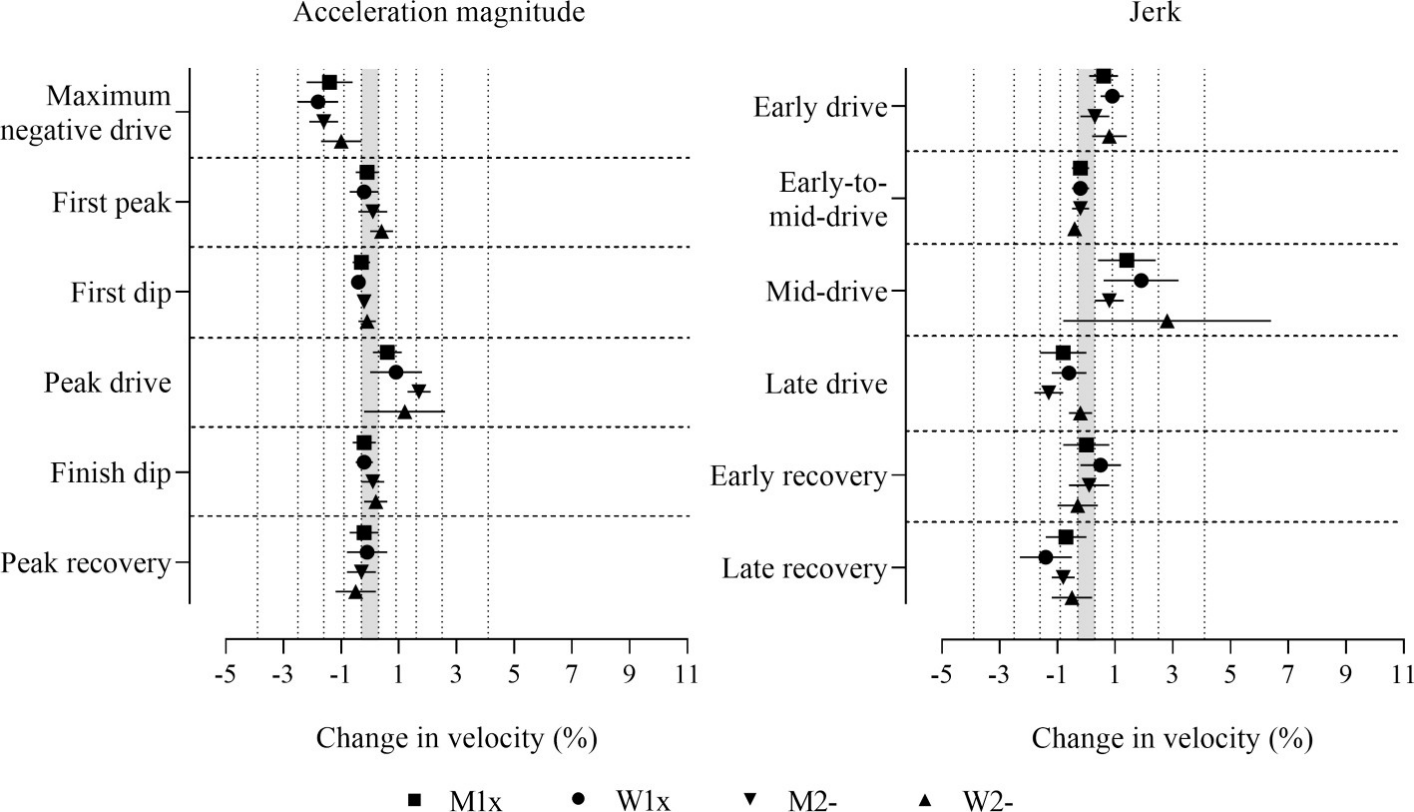
Change in boat velocity for a change in predictor variables of two within-crew standard deviations with adjustment for power output and stroke rate. Data are mean (%) and 90% compatibility intervals. The shaded grey area covers trivial values (values within the smallest substantial change thresholds, -0.3 to 0.3%). Vertical dotted lines delineate threshold magnitudes of small (±0.3%), moderate (±0.9%), large (±1.6%), very large (±2.5%), and extremely large (4.1, -3.9%). Effects with compatibility intervals that do not enter the grey area are decisively (very likely or most likely) substantial. Effects with compatibility intervals that end within the grey area have adequate precision (possibly or likely substantial or trivial).

The adjustment of power and stroke rate reduced the magnitudes of most effects to the ranges of trivial to moderate (Fig 5 and S3 Table). Negative effects for maximum negative drive acceleration were decisively substantial in all boat classes. Positive effects for jerk in the early drive and the mid-drive, and peak drive acceleration magnitude, and negative effects for jerk in the late drive and the late recovery were decisively substantial in some boat classes (those with 90% compatibility intervals entirely in substantial values in Fig 5). Measures where the effects had adequate precision but were only possibly or likely substantial, include positive effects in some boat classes for jerk in the early drive, the mid-drive and the early recovery, first peak and peak drive acceleration magnitudes, and negative effects in some boat classes for jerk in the early-to-mid-drive, the late drive and the late recovery, first dip and peak recovery acceleration magnitudes (those with more than half of the 90% compatibility intervals overlapping substantial values in Fig 5). First dip acceleration magnitude in men’s pairs was the only decisively trivial effect. Trivial observed effects that had adequate precision, where the true magnitudes were possibly or likely trivial were found in some boat classes for jerk in the early-to-mid-drive, first peak, first dip, and finish dip acceleration magnitudes (those with less than half of the 90% compatibility intervals overlapping substantial values in Fig 5).

**Fig 6:**
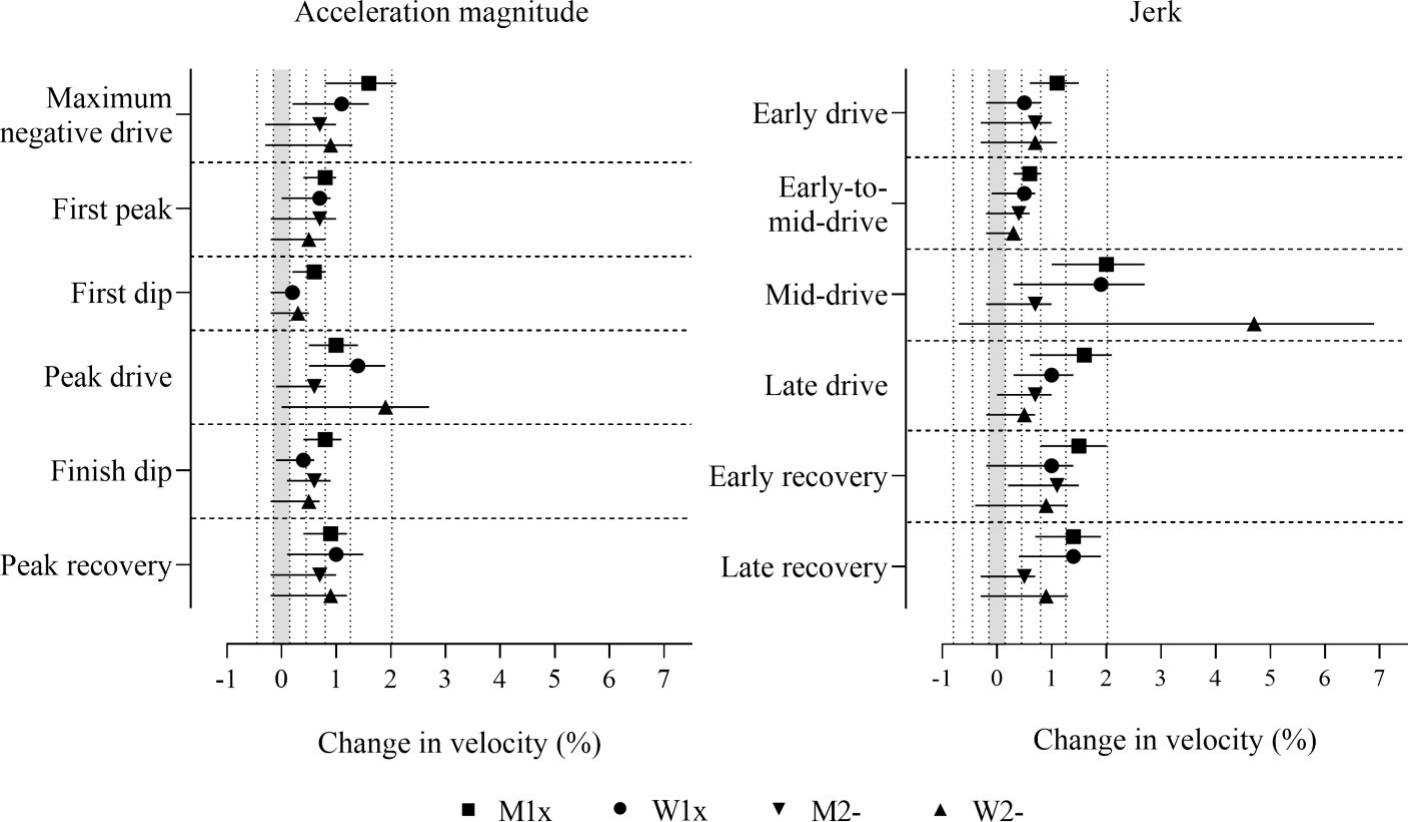
Differences between crews in the effects of the predictor variables with adjustment for power output and stroke rate. Data are SD (%) and 90% compatibility intervals. The shaded grey area covers trivial values (values within the smallest substantial difference thresholds, -0.15 to 0.15%). Vertical dotted lines delineate threshold magnitudes of small (±0.15), moderate (±0.45), large (+0.80), very large (+1.3), and extremely large (+2.0). Effects with compatibility intervals that do not enter the grey area are decisively (very likely or most likely) substantial. Effects with compatibility intervals that end within the grey area have adequate precision (possibly or likely substantial or trivial).

Between-crew differences in the effect of predictor variables on velocity before adjustment were mostly moderate to very large in magnitude across all boat classes and were decisively substantial for most predictors in men’s singles, and for some predictors in women’s singles (refer to S4 Table). Precision was inadequate for between-crew differences in most predictors for men’s and women’s pairs. With adjustment for stroke rate (S5 Table) between-crew differences increased in magnitude to the range of large to extremely large, with decisively substantial differences observed for all predictors in men’s and women’s singles and for most predictors in men’s pairs. Precision remained inadequate for between-crew differences in most predictors for women’s pairs (refer to S5 Table). After adjustment for stroke rate and power (Fig 6 and S6 Table) between-crew differences were of similar magnitude and precision as those before adjustment, as described above and in S4 Table.

## Discussion

Boat acceleration profiles are commonly used to provide feedback on rowing technique; however, current interpretation of acceleration profiles is largely informed by experiential knowledge given research in this area is limited to the drive phase and findings are conflicting. This investigation of relationships with performance for variables describing the boat acceleration profile provides empirical evidence for the evaluation of acceleration profiles in rowing. In this study, six variables of acceleration magnitude and jerk per stroke were assessed in relation to boat velocity during 2000-m racing in singles and pairs. Decisively substantial effects observed in all boat classes for maximum negative drive and peak drive acceleration magnitudes, and for jerk in the early drive, the mid-drive (excluding women’s pairs), and the late drive after adjustment for stroke rate reveal aspects of the acceleration profile relating to performance. Effect magnitudes were reduced after adjustment for stroke rate and power in most variables, illustrating their mediating effect on measures of boat acceleration.

The adjustment of stroke rate was required given its effect on boat acceleration [1, 6], but also to provide insight into potential strategies for improving performance beyond increasing stroke rate. Effect magnitudes either increased or decreased after adjustment for stroke rate, depending on the predictor assessed. The increased effect magnitudes observed for maximum negative drive and peak drive acceleration magnitude, and jerk in the late drive likely reflect the effect of force applied during the drive on boat acceleration, given the strong relationship (R^2^ = 0.904) between the force curve and boat acceleration profile during the drive phase [3]. Faster rower COM velocities during the drive would likely improve force application (via higher oar angular velocities) and therefore peak drive acceleration magnitude, however, the adjustment for stroke rate can be expected to also account for the effect of oar angular velocity on force production, and therefore would not contribute to the increased effect magnitudes for peak drive acceleration with adjustment for stroke rate. Reduced effect magnitudes when adjusted for stroke rate describe the mediating effect of stroke rate on peak recovery acceleration magnitude and jerk in the early drive, the early recovery and the late recovery, whereby higher stroke rates increase the magnitudes of these variables. The mediating effect of stroke rate is likely related to faster rower COM movement during the recovery, given most of these variables occurred during the recovery phase, and increased stroke rate is largely achieved via shorter recovery phase duration [16, 17].

Relationships between the acceleration measures assessed and boat velocity with adjustment for both power and stroke rate may provide the most meaningful practical impact for coaches and sport scientists in the interpretation of acceleration profiles (those effects presented in Fig 5 and S3 Table), as the effects on velocity are not attributable to the power applied or the stroke rate performed, but rather reflect additional performance improvements. The adjustment for power output informs measures of acceleration associated with enhancements in rowing velocity for the same given power output. Specifically, deeper maximum negative drive magnitudes, greater positive peak drive magnitudes, greater jerk magnitudes in the early and mid-drive, and more negative jerk magnitudes in the late drive and late recovery phases are measures associated with further benefits to performance. Moreover, the large to very large effect magnitudes for both stroke rate and power on boat velocity established elsewhere [8] demonstrate the need for the adjustment of both stroke rate and power output when assessing relationships between biomechanical variables and velocity. The additional adjustment for power with adjustment for stroke rate reduced effect magnitudes resulting in the loss of precision for many effects, revealing the mediating effect of power output on most of the variables assessed. The reduction of effect magnitudes after the additional adjustment for power for jerk in the late drive, maximum negative drive acceleration magnitude, and peak drive acceleration magnitude suggest that the effects observed for these variables prior to the adjustment for power simply reflect the positive relationship between power and velocity, whereby faster velocities associated with greater magnitudes of these variables are explained by higher power outputs.

The negative effects for maximum negative drive acceleration magnitude and jerk in the late recovery after adjustment for stroke rate, and adjustment for stroke rate and power, illustrate the transition from late recovery to the catch and early drive phases of the rowing stroke as having an important association with velocity. The late recovery, catch and early drive phases of the stroke require highly technical movement coordination occurring over a very short time period (approximately 0.4 s at a stroke rate of 32 min^-1^), consisting of rower COM change of direction, blade placement and force application [1]. Although rower COM acceleration and force applied at the footplate were not assessed in the current study, the negative effects for jerk in the late recovery may relate to a delayed negative acceleration of rower COM in the late recovery, requiring greater resultant sternward directed force at the footplate over a shorter time period. A larger resultant force at the footplate applied over a shorter time period can be expected to correspond to deeper (i.e., less positive) maximum negative drive accelerations occurring with effective blade placement at the catch. An advantageous performance effect of delayed negative acceleration of rower COM during the recovery has not been investigated, however has been implemented by elite rowers and coaches [18; authors’ observations]. An example of this includes the finish pause adopted by many international crews at low stroke rates, which encourages rowers to move with the boat on the recovery, rather than slowing themselves down into the front turn. However, further research investigating relationships between maximum negative drive acceleration, jerk between peak recovery acceleration and maximum negative drive acceleration, force applied at the footplate, and rower COM acceleration during the recovery phase is required to confirm any effect on performance of delayed negative acceleration of rower COM during the recovery.

The positive effects for first peak and peak drive acceleration magnitudes, and jerk in the early drive and the mid-drive after adjustment for stroke rate, and for most of these variables after adjustment for stroke rate and power, likely relate to force application during the drive phase. The boat acceleration profile during the drive phase reflects force curve shape [3], with force application during the drive demonstrating extremely large positive relationships with rowing performance [8]. The positive effects for jerk in the early drive and the mid-drive may reflect rate of force development, which has a positive relationship with rowing performance [8, 19] and is proposed to increase the impulse and subsequent boat velocity achieved [20, 21]. Therefore, enhanced propulsive force application during the drive is expected to result in the achievement of higher first peak and peak drive acceleration magnitudes, and increased jerk in the early drive and in the mid-drive.

The between-crew differences in predictors after adjustment for stroke rate, demonstrate the crew-specific nature of relationships between velocity and measures of boat acceleration. For predictors with adequate precision in both their mean effect and between-crew differences, a change in the predictor is expected to be associated with a change in performance in the same direction in most crews, however is best investigated on an individual basis to determine the magnitude of the association for a specific crew. As such, individual-based analyses can reveal areas of the boat acceleration profile associated with improved performance in a particular crew that are not evident from the mean effects for the cohort assessed. The random effect solutions for crew identity in the current model present differences from the mean modifying effect for each crew, allowing the assessment of crew-specific relationships, and therefore the provision of individualised feedback on boat acceleration profiles.

### Practical applications

- Stroke rate and power have mediating effects on most measures of boat acceleration magnitude and jerk, and should be considered when assessing the boat acceleration profile.
- Deeper (i.e., less positive) maximum negative drive accelerations and greater jerk in the late recovery are associated with faster velocities, revealing the late recovery as an important area for technical focus.
- Improved performance is also associated with greater peak drive acceleration magnitudes, and jerk in the early drive and the mid-drive, likely reflecting force application during the drive.
- Relationships between some measures of the boat acceleration profile and velocity differ between crews and are best assessed on an individual basis.

## Conclusion

Deeper maximum negative drive accelerations and greater jerk in the late recovery are related to faster velocities, likely reflecting delayed rower centre-of-mass negative acceleration in preparation for the catch. Peak drive and first peak accelerations, and jerk between the first dip in acceleration during the drive and peak drive acceleration are associated with improved performance, likely reflecting propulsive force during the drive. Practitioners should consider accounting for differences in power output and stroke rate (such as statistically or during the collection of data) when assessing boat acceleration profiles in rowing due to the mediating effect of these variables.

## Supporting information

S1 Table Change in boat velocity for a change in predictor variables of two within-crew standard deviations without adjustment in the four boat classes. Data are mean (%), ±90% compatibility limits, with observed magnitude and p values for non-inferiority and non-superiority tests (p–/p+).
(DOCX)

S2 Table Change in boat velocity for a change in predictor variables of two within-crew standard deviations with adjustment for stroke rate in the four boat classes. Data are mean (%), ±90% compatibility limits, with observed magnitude and p values for non-inferiority and non-superiority tests (p_–_/p_+_).
(DOCX)

S3 Table Change in boat velocity for a change in predictor variables of two within-crew standard deviations with adjustment for stroke rate and power in the four boat classes. Data are SD (%), ±90% compatibility limits, with observed magnitude and p values for non-inferiority and non-superiority tests (p_–_/p_+_).
(DOCX)

S4 Table Differences between crews in the effects of the predictor variables before adjustment (in S1 Table) in the four boat classes. Data are SD (%), ±90% compatibility limits (approximate), with observed magnitude and p values for non-inferiority and non-superiority tests (p_–_/p_+_).
(DOCX)

S5 Table Differences between crews in the effects of the predictor variables with adjustment for stroke rate (in S2 Table) in the four boat classes. Data are SD (%), ±90% compatibility limits (approximate), with observed magnitude and p values for non-inferiority and non-superiority tests (p_–_/p_+_).
(DOCX)

S6 Table Differences between crews in the effects of the predictor variables with adjustment for stroke rate and power (in S3 Table) in the four boat classes. Data are SD (%), ±90% compatibility limits (approximate), with observed magnitude and p values for non-inferiority and non-superiority tests (p_–_/p_+_).
(DOCX)
