# Supplementary material for: Relationships between measures of boat acceleration and performance in rowing, with and without controlling for stroke rate and power output
Source: PLoS One. 2021 Aug 20;16(8):e0249122. doi: 10.1371/journal.pone.0249122 (PMC8378734; doi:10.1371/journal.pone.0249122)
Supplement: S1 Table — Data are mean (%), ±90% compatibility limits, with observed magnitude and p values for non-inferiority and non-superiority tests (p–/p+). (DOCX) [file pone.0249122.s001.docx]

| **S1 Table**. **Change in boat velocity for a change in predictor variables of two within-crew standard deviations without adjustment in the four boat classes.** Data are mean (%), ±90% compatibility limits, with observed magnitude and p values for non-inferiority and non-superiority tests (p_–_/p_+_). | | | | |
| --- | --- | --- | --- | --- |
|  | Single sculls | | Coxless pairs | |
|  | Men  (M1x) | Women (W1x) | Men  (M2-) | Women (W2-) |
| **Acceleration magnitude** | | | | |
| Maximum negative drive | **-2.3, ±0.7;**  **large******  >0.999/<0.001 | **-2.4, ±0.7;**  **large******  0.999/<0.001 | **-2.1, ±0.6;**  **large******  >0.999/<0.001 | **-2.2, ±0.8;**  **large******  0.997/0.001 |
| First peak | 0.4, ±0.4;  small*^0^  0.006/0.61 | 0.1, ±0.5;  trivial  0.08/0.31 | **0.6, ±0.5;**  **small****  0.004/0.84 | **1.1, ±0.5;**  **mod*****  0.001/0.99 |
| First dip | **-0.3, ±0.3;**  **trivial^0^***  0.48/0.002 | **-0.3, ±0.3;**  **small*^0^**  0.53/0.001 | **-0.1, ±0.1;**  **trivial^0000^**  0.004/<0.001 | -0.0, ±0.3;  trivial^00^  0.06/0.04 |
| Peak drive | **0.8, ±0.6;**  **small****  0.004/0.91 | 1.2, ±0.9;  mod**  0.01/0.94 | **1.5, ±0.3;**  **mod******  <0.001/>0.999 | 1.6, ±1.4;  large**  0.02/0.94 |
| Finish dip | -0.0, ±0.4;  trivial  0.14/0.11 | **-0.2, ±0.2;**  **trivial^0^***  0.28/0.001 | 0.1, ±0.5;  trivial  0.08/0.22 | 0.4, ±0.3;  small*^0^  0.001/0.64 |
| Peak recovery | **0.9, ±0.5;**  **small*****  0.001/0.97 | 1.0, ±0.8;  mod**  0.01/0.92 | 0.5, ±0.5;  small**  0.006/0.83 | 0.9, ±0.8;  mod**  0.01/0.90 |
| **Jerk** | | | | |
| Early drive phase | **1.4, ±0.5;**  **mod******  <0.001/0.999 | **1.5, ±0.5;**  **mod******  <0.001/0.999 | **1.1, ±0.5;**  **mod*****  0.001/0.99 | **1.7, ±0.8;**  **large*****  0.001/0.99 |
| Early-to-mid-drive phase | **-0.5, ±0.3;**  **small****  0.83/0.001 | -0.4, ±0.4;  small*^0^  0.60/0.01 | **-0.4, ±0.3;**  **small****  0.81/<0.001 | **-0.7, ±0.4;**  **small*****  0.96/0.002 |
| Mid-drive phase | **1.8, ±1.1;**  **large*****  0.01/0.99 | **2.5, ±1.4;**  **large*****  0.004/0.99 | **0.9, ±0.5;**  **mod*****  0.002/0.98 | 3.6, ±4.4;  v.large  0.03/0.91 |
| Late drive phase | -1.0, ±1.0;  mod**  0.89/0.02 | -0.8, ±0.8;  small**  0.86/0.02 | **-1.3, ±0.5;**  **mod*****  0.99/<0.001 | -0.2, ±0.4;  trivial^0^*  0.32/0.03 |
| Early recovery phase | **1.0, ±0.7;**  **mod****  0.004/0.95 | **1.4, ±0.9;**  **mod*****  0.004/0.98 | 0.7, ±0.7;  small**  0.02/0.84 | 1.2, ±1.3;  mod**  0.03/0.89 |
| Late recovery phase | **-1.9, ±0.7;**  **large******  0.999/<0.001 | **-2.3, ±1.0;**  **large******  0.996/0.001 | **-1.4, ±0.4;**  **mod******  0.999/0.001 | **-2.0, ±0.7;**  **large******  0.997/0.001 |
| M1x, men’s singles; W1x, women’s singles; M2-, men’s coxless pairs; W2- women’s coxless pairs.  Number of crews: 14, 9, 9 and 7 respectively.  Number of races: 25, 18, 18, 13 respectively.  Scale of magnitudes: <0.3%, trivial; 0.3-0.9%, small; 0.9-1.6%, moderate (mod); 1.6-2.5%, large; 2.5-4.1%, very large (v.large); >4.1%, extremely large (e.large).  Reference-Bayesian likelihoods of substantial change: *possibly; **likely; ***very likely, ****most likely.  *** and **** indicate rejection of the non-superiority or non-inferiority hypothesis (p_N-_ or p_N+_ <0.05 and <0.005 respectively).  Reference-Bayesian likelihoods of trivial change: ^0^possibly; ^00^likely; ^000^very likely, ^0000^most likely.  Likelihoods are not shown for effects with inadequate precision at the 90% level (failure to reject any hypotheses: p>0.05).  Effects in **bold** have adequate precision at the 99% level (p<0.005). | | | | |
