# Supplementary material for: Relationships between measures of boat acceleration and performance in rowing, with and without controlling for stroke rate and power output
Source: PLoS One. 2021 Aug 20;16(8):e0249122. doi: 10.1371/journal.pone.0249122 (PMC8378734; doi:10.1371/journal.pone.0249122)
Supplement: S2 Table — Data are mean (%), ±90% compatibility limits, with observed magnitude and p values for non-inferiority and non-superiority tests (p–/p+). (DOCX) [file pone.0249122.s002.docx]

| **S2 Table**. **Change in boat velocity for a change in predictor variables of two within-crew standard deviations with adjustment for stroke rate in the four boat classes.** Data are mean (%), ±90% compatibility limits, with observed magnitude and p values for non-inferiority and non-superiority tests (p_–_/p_+_). | | | | |
| --- | --- | --- | --- | --- |
|  | Single sculls | | Coxless pairs | |
|  | Men  (M1x) | Women (W1x) | Men  (M2-) | Women (W2-) |
| **Acceleration magnitude** | | | | |
| Maximum negative drive | **-3.4, ±0.5;**  **v.large******  >0.999/<0.001 | **-3.6, ±1.1;**  **v.large******  >0.999/<0.001 | **-3.7, ±1.4;**  **v.large******  0.999/<0.001 | **-3.1, ±1.1;**  **v.large******  0.999/<0.001 |
| First peak | 0.2, ±0.7;  trivial  0.10/0.39 | 1.2, ±1.1;  mod**  0.02/0.92 | 0.9, ±1.0;  small**  0.03/0.96 | 2.0, ±1.5;  large***  0.01/0.96 |
| First dip | 0.2, ±0.6;  trivial  0.06/0.43 | -0.6, ±0.5;  small**  0.88/0.003 | -0.1, ±0.4;  trivial  0.22/0.06 | -0.0, ±0.6;  trivial  0.22/0.18 |
| Peak drive | **1.8, ±0.8;**  **large******  <0.001/0.999 | **2.6, ±0.7;**  **v.large******  <0.001/>0.999 | **3.3, ±1.0;**  **v.large******  <0.001/>0.999 | **3.4, ±1.9;**  **v.large*****  0.004/0.99 |
| Finish dip | **-0.6, ±0.5;**  **small****  0.83/0.004 | -0.2, ±0.7;  trivial  0.36/0.12 | 0.4, ±0.6;  small*^0^  0.04/0.59 | 0.7, ±0.6;  small**  0.01/0.84 |
| Peak recovery | -0.2, ±0.7;  trivial  0.37/0.13 | 0.5, ±1.1;  small  0.12/0.60 | 0.2, ±1.3;  trivial  0.23/0.46 | -0.5, ±1.6;  small  0.59/0.18 |
| **Jerk** | | | | |
| Early drive phase | **1.7, ±0.6;**  **large******  <0.001/>0.999 | **2.5, ±1.0;**  **v.large******  <0.01/0.999 | 1.6, ±1.2;  mod***  0.01/0.96 | **2.3, ±1.2;**  **large*****  0.002/0.99 |
| Early-to-mid-drive phase | -0.2, ±0.7;  trivial  0.35/0.12 | **-1.3, ±0.7;**  **mod*****  0.98/0.002 | -0.6, ±0.7;  small**  0.78/0.02 | **-1.2, ±0.8;**  **mod*****  0.97/0.005 |
| Mid-drive phase | 1.8, ±1.4;  large***  0.009/0.96 | **3.1, ±1.2;**  **v.large******  <0.001/0.995 | **1.5, ±0.7;**  **mod*****  <0.001/0.999 | 4.9, ±5.7;  e.large  0.06/0.92 |
| Late drive phase | **-1.8, ±1.0;**  **large*****  0.99/0.002 | **-1.3, ±0.8;**  **mod*****  0.98/0.003 | **-2.5, ±0.9;**  **v.large******  0.999/<0.001 | **-1.2, ±0.7;**  **mod*****  0.98/0.002 |
| Early recovery phase | 0.4, ±0.8;  small  0.06/0.059 | 0.8, ±1.1;  small**  0.05/0.79 | 0.5, ±0.9;  small  0.07/0.67 | -0.3, ±1.5;  small  0.50/0.23 |
| Late recovery phase | -1.4, ±1.0;  mod***  0.95/0.008 | -2.6, ±1.9;  v.large***  0.97/0.01 | -1.7, ±1.8;  large**  0.91/0.04 | -0.7, ±1.2;  small  0.75/0.07 |
| M1x, men’s singles; W1x, women’s singles; M2-, men’s coxless pairs; W2- women’s coxless pairs.  Number of crews: 14, 9, 9 and 7 respectively.  Number of races: 25, 18, 18, 13 respectively.  Scale of magnitudes: <0.3%, trivial; 0.3-0.9%, small; 0.9-1.6%, moderate (mod); 1.6-2.5%, large; 2.5-4.1%, very large (v.large); >4.1%, extremely large (e.large).  Reference-Bayesian likelihoods of substantial change: *possibly; **likely; ***very likely, ****most likely.  *** and **** indicate rejection of the non-superiority or non-inferiority hypothesis (p_N-_ or p_N+_ <0.05 and <0.005 respectively).  Reference-Bayesian likelihoods of trivial change: ^0^possibly; ^00^likely; ^000^very likely, ^0000^most likely.  Likelihoods are not shown for effects with inadequate precision at the 90% level (failure to reject any hypotheses: p>0.05).  Effects in **bold** have adequate precision at the 99% level (p<0.005). | | | | |
