# Supplementary material for: Relationships between measures of boat acceleration and performance in rowing, with and without controlling for stroke rate and power output
Source: PLoS One. 2021 Aug 20;16(8):e0249122. doi: 10.1371/journal.pone.0249122 (PMC8378734; doi:10.1371/journal.pone.0249122)
Supplement: S3 Table — Data are SD (%), ±90% compatibility limits, with observed magnitude and p values for non-inferiority and non-superiority tests (p–/p+). (DOCX) [file pone.0249122.s003.docx]

| **S3 Table**. **Change in boat velocity for a change in predictor variables of two within-crew standard deviations with adjustment for stroke rate and power in the four boat classes.** Data are SD (%), ±90% compatibility limits, with observed magnitude and p values for non-inferiority and non-superiority tests (p_–_/p_+_). | | | | |
| --- | --- | --- | --- | --- |
|  | Single sculls | | Coxless pairs | |
|  | Men  (M1x) | Women (W1x) | Men  (M2-) | Women (W2-) |
| **Acceleration magnitude** | | | | |
| Maximum negative drive | **-1.4, ±0.8;**  **mod*****  0.98/0.001 | **-1.8, ±0.7;**  **large******  0.997/<0.001 | **-1.6, ±0.5;**  **mod******  0.999/0.001 | **-1.0, ±0.7;**  **mod*****  0.96/0.005 |
| First peak | -0.1, ±0.4;  trivial  0.19/0.06 | -0.2, ±0.5;  trivial^0^*  0.38/0.04 | 0.1, ±0.5;  trivial  0.66/0.26 | 0.4, ±0.4;  small*^0^  0.008/0.70 |
| First dip | **-0.3, ±0.3;**  **small*^0^**  0.52/0.002 | **-0.4, ±0.2;**  **small****  0.76/<0.001 | **-0.2, ±0.1;**  **trivial^000^**  0.02/<0.001 | -0.1, ±0.3;  trivial^00^  0.16/0.02 |
| Peak drive | 0.6, ±0.5;  small**  0.006/0.83 | 0.9, ±0.9;  mod**  0.02/0.90 | **1.7, ±0.4;**  **large******  <0.001/>0.999 | 1.2, ±1.4;  mod**  0.04/0.85 |
| Finish dip | -0.2, ±0.4;  trivial^0^*  0.34/0.03 | -0.2, ±0.3;  trivial^0^*  0.31/0.004 | 0.1, ±0.4;  trivial  0.08/0.19 | 0.2, ±0.4;  trivial^0^*  0.02/0.37 |
| Peak recovery | -0.2, ±0.5;  trivial  0.31/0.07 | -0.1, ±0.7;  trivial  0.29/0.16 | -0.3, ±0.5;  small*^0^  0.51/0.03 | -0.5, ±0.7;  small*  0.75/0.03 |
| **Jerk** | | | | |
| Early drive phase | 0.6, ±0.5;  small**  0.009/0.82 | **0.9, ±0.4;**  **mod*****  <0.001/0.99 | 0.3, ±0.5;  small*  0.03/0.53 | 0.8, ±0.6;  small**  0.007/0.91 |
| Early-to-mid-drive phase | -0.2, ±0.3;  trivial^0^*  0.65/0.009 | -0.2, ±0.3;  trivial^0^*  0.38/0.001 | -0.2, ±0.3;  trivial^0^*  0.28/0.01 | -0.4, ±0.2;  small*^0^  0.66/0.001 |
| Mid-drive phase | 1.4, ±1.0;  mod***  0.008/0.96 | 1.9, ±1.3;  large***  0.007/0.98 | **0.8, ±0.5;**  **small****  0.003/0.94 | 2.8, ±3.6;  v.large  0.07/0.89 |
| Late drive phase | -0.8, ±0.8;  small**  0.86/0.02 | -0.6, ±0.6;  small**  0.83/0.01 | **-1.3, ±0.5;**  **mod******  0.997/<0.001 | -0.2, ±0.4;  trivial^0^*  0.30/0.03 |
| Early recovery phase | 0.0, ±0.8;  trivial  0.23/0.28 | 0.5, ±0.7;  small*^0^  0.03/0.69 | 0.1, ±0.7;  trivial  0.16/0.34 | -0.3, ±0.7;  small  0.53/0.08 |
| Late recovery phase | -0.7, ±0.7;  small*  0.82/0.02 | **-1.4, ±0.9;**  **mod*****  0.97/0.004 | **-0.8, ±0.4;**  **small*****  0.96/<0.001 | -0.5, ±0.7;  small*  0.72/0.03 |
| M1x, men’s singles; W1x, women’s singles; M2-, men’s coxless pairs; W2- women’s coxless pairs.  Number of crews: 14, 9, 9 and 7 respectively.  Number of races: 25, 18, 18, 13 respectively.  Scale of magnitudes: <0.3%, trivial; 0.3-0.9%, small; 0.9-1.6%, moderate (mod); 1.6-2.5%, large; 2.5-4.1%, very large (v.large); >4.1%, extremely large (e.large).  Reference-Bayesian likelihoods of substantial change: *possibly; **likely; ***very likely, ****most likely.  *** and **** indicate rejection of the non-superiority or non-inferiority hypothesis (p_N-_ or p_N+_ <0.05 and <0.005 respectively).  Reference-Bayesian likelihoods of trivial change: ^0^possibly; ^00^likely; ^000^very likely, ^0000^most likely.  Likelihoods are not shown for effects with inadequate precision at the 90% level (failure to reject any hypotheses: p>0.05).  Effects in **bold** have adequate precision at the 99% level (p<0.005). | | | | |
