# Supplementary material for: Relationships between measures of boat acceleration and performance in rowing, with and without controlling for stroke rate and power output
Source: PLoS One. 2021 Aug 20;16(8):e0249122. doi: 10.1371/journal.pone.0249122 (PMC8378734; doi:10.1371/journal.pone.0249122)
Supplement: S5 Table — Data are SD (%), ±90% compatibility limits (approximate), with observed magnitude and p values for non-inferiority and non-superiority tests (p–/p+). (DOCX) [file pone.0249122.s005.docx]

| **S5 Table. Differences between crews in the effects of the predictor variables with adjustment for stroke rate (in S2 Table) in the four boat classes**. Data are SD (%), ±90% compatibility limits (approximate), with observed magnitude and p values for non-inferiority and non-superiority tests (p_–_/p_+_). | | | | |
| --- | --- | --- | --- | --- |
|  | Single sculls | | Coxless pairs | |
|  | Men  (M1x) | Women (W1x) | Men  (M2-) | Women (W2-) |
| **Acceleration magnitude** | | | | |
| Maximum negative drive | 1.0, ±0.5;  large***  0.02/0.98 | 1.8, ±0.9;  v.large***  0.03/0.97 | 2.2, ±1.1;  e.large***  0.03/0.97 | 1.4, ±1.2;  v.large  0.06/0.93 |
| First peak | 1.3, ±0.5;  v.large***  0.008/0.99 | 1.7, ±0.8;  v.large***  0.03/0.97 | 1.6, ±0.8;  v.large***  0.03/0.97 | 2.0, ±1.4;  v.large**  0.05/0.95 |
| First dip | 1.2, ±0.4;  large***  0.009/0.99 | 0.7, ±0.4;  mod***  0.06/0.93 | 0.7, ±0.4;  mod**  0.04/0.95 | 0.8, ±0.7;  large  0.06/0.93 |
| Peak drive | 1.6, ±0.6;  v.large***  0.007/0.99 | 1.0, ±0.5;  large***  0.05/0.95 | 1.5, ±0.7;  v.large***  0.03/0.96 | 2.4, ±1.6;  e.large***  0.05/0.95 |
| Finish dip | 1.0, ±0.4;  large***  0.08/0.99 | 1.0, ±0.6;  large***  0.03/0.96 | 1.0, ±0.5;  large***  0.03/0.96 | 0.8, ±0.7;  large  0.05/0.94 |
| Peak recovery | 1.4, ±0.6;  v.large***  0.01/0.99 | 1.7, ±0.9;  v.large***  0.03/0.97 | 2.0, ±1.0;  v.large***  0.03/0.97 | 2.1, ±1.6;  e.large  0.05/0.95 |
| **Jerk** | | | | |
| Early drive phase | 1.1, ±0.5;  large***  0.01/0.98 | 1.5, ±0.8;  v.large***  0.03/0.94 | 1.8, ±1.0;  v.large***  0.03/0.97 | 1.4, ±1.2;  v.large  0.06/0.94 |
| Early-to-mid-drive phase | 1.3, ±0.5;  v.large***  0.01/0.99 | 1.0, ±0.6;  large***  0.04/0.96 | 1.1, ±0.6;  large***  0.03/0.97 | 1.0, ±0.8;  large  0.05/0.94 |
| Mid-drive phase | 2.8, ±1.1;  e.large***  0.008/0.99 | 1.9, ±1.0;  v.large***  0.03/0.97 | 1.0, ±0.5;  large***  0.03/0.97 | 7.3, ±4.9;  e.large***  0.05/0.96 |
| Late drive phase | 2.0, ±0.9;  e.large***  0.02/0.94 | 1.2, ±0.7;  large***  0.03/0.96 | 1.4, ±0.7;  v.large***  0.03/0.97 | 0.8, ±0.7;  large***  0.05/0.94 |
| Early recovery phase | 1.5, ±0.6;  v.large*** | 1.7, ±0.9;  v.large***  0.01/0.99 | 1.7, ±0.9;  v.large***  0.03/0.97 | 1.9, ±1.8;  v.large  0.08/0.92 |
| Late recovery phase | 2.1, ±0.9;  e.large***  0.01/0.99 | 3.1, ±1.5;  e.large***  0.03/0.98 | 2.9, ±1.5;  e.large***  0.03/0.97 | 1.5, ±1.4;  v.large  0.07/0.92 |
| M1x, men’s singles; W1x, women’s singles; M2-, men’s coxless pairs; W2- women’s coxless pairs.  Number of crews: 14, 9, 9 and 7 respectively.  Number of races: 25, 18, 18, 13 respectively.  Scale of magnitudes: <0.15%, trivial; 0.15-0.45%, small; 0.45-0.8%, moderate (mod); 0.8-1.26%, large; 1.26-2.02%, very large (v.large); >2.02%, extremely large (e.large).  Reference-Bayesian likelihoods of substantial change: *possibly; **likely; ***very likely, ****most likely.  *** and **** indicate rejection of the non-superiority or non-inferiority hypothesis (p_N-_ or p_N+_ <0.05 and <0.005 respectively).  Likelihoods are not shown for effects with inadequate precision at the 90% level (failure to reject any hypotheses: p>0.05).  Effects in **bold** have adequate precision at the 99% level (p<0.005). | | | | |
